# Longitudinal assessment of antibiotic resistance gene profiles in gut microbiomes of infants at risk of eczema

**DOI:** 10.1186/s12879-020-05000-y

**Published:** 2020-04-28

**Authors:** Evelyn Xiu Ling Loo, Amanda Zain, Gaik Chin Yap, Rikky W. Purbojati, Daniela I. Drautz-Moses, Yan Qing Koh, Yap Seng Chong, Kok Hian Tan, Peter D. Gluckman, Fabian Yap, Johan Gunnar Eriksson, Elizabeth Tham, Lynette Pei-chi Shek, Staffan Kjelleberg, Stephan C. Schuster, Ritu Banerjee, Bee Wah Lee

**Affiliations:** 1 Singapore Institute for Clinical Sciences (SICS), Agency for Science, Technology and Research (A*STAR), Singapore, Singapore; 2 Department of Paediatrics, Yong Loo Lin School of Medicine, National University of Singapore, Singapore, Singapore; 3 Khoo Teck Puat-National University Children’s Medical Institute, National University Hospital, National University Health System, Singapore, Singapore; 4 Singapore Centre For Environmental Life Sciences Engineering (SCELSE), Nanyang Technological University, Singapore, Singapore; 5 Department of Obstetrics & Gynaecology, Yong Loo Lin School of Medicine, National University of Singapore and National University Health System, Singapore, Singapore; 6 Department of Maternal Fetal Medicine, KK Women’s and Children’s Hospital (KKH), Singapore, Singapore; 7 Liggins Institute, University of Auckland, Auckland, New Zealand; 8 Department of Endocrinology KK Women’s and Children’s Hospital (KKH), Singapore, Singapore; 9 Vanderbilt University Medical Center, Nashville, TN USA

**Keywords:** Antibiotic resistance genes, Resistome, Infancy, Birth cohort, ESBL producing *Enterobacteriaceae*

## Abstract

**Background:**

While there is increasing knowledge about the gut microbiome, the factors influencing and the significance of the gut resistome are still not well understood. Infant gut commensals risk transferring multidrug-resistant antibiotic resistance genes (ARGs) to pathogenic bacteria. The rapid spread of multidrug-resistant pathogenic bacteria is a worldwide public health concern. Better understanding of the naïve infant gut resistome may build the evidence base for antimicrobial stewardship in both humans and in the food industry. Given the high carriage rate of extended spectrum beta-lactamase (ESBL)-producing *Enterobacteriaceae* in Asia, we aimed to evaluate community prevalence, dynamics, and longitudinal changes in antibiotic resistance gene (ARG) profiles and prevalence of ESBL-producing *E. coli* and *K. pneumoniae* in the intestinal microbiome of infants participating in the Growing Up in Singapore Towards Healthy Outcomes (GUSTO) study, a longitudinal cohort study of pregnant women and their infants.

**Methods:**

We analysed ARGs in the first year of life among 75 infants at risk of eczema who had stool samples collected at multiple timepoints using metagenomics.

**Results:**

The mean number of ARGs per infant increased with age. The most common ARGs identified confer resistance to aminoglycoside, beta-lactam, macrolide and tetracycline antibiotics; all infants harboured these antibiotic resistance genes at some point in the first year of life. Few ARGs persisted throughout the first year of life. Beta-lactam resistant *Escherichia coli* and *Klebsiella pneumoniae* were detected in 4 (5.3%) and 32 (42.7%) of subjects respectively.

**Conclusion:**

In this longitudinal cohort study of infants living in a region with high endemic antibacterial resistance, we demonstrate that majority of the infants harboured several antibiotic resistance genes in their gut and showed that the infant gut resistome is diverse and dynamic over the first year of life.

## Background

The rapid spread of multidrug-resistant pathogenic bacteria that are no longer susceptible to treatment with common antibiotics is a global public health concern. The lack of efficacy of currently available antibiotics and the paucity of new antibiotic development may potentially result in a situation akin to the pre-antibiotic era where minor infections may become life-threatening conditions [1–3].

The human gastrointestinal tract hosts a diversity of microbiota which are responsible for important functions such as stimulation of intestinal angiogenesis, protection against cell injury and regulation of fat storage. However, the gut microbiota is a reservoir for antibiotic resistance genes (ARGs). ARGs have been identified in the intestinal microbiomes from adults as well as infants as early as the first month of life [4–9]. Alarmingly, ARGs in commensal human gut microbiota can be transferred to pathogenic strains of bacteria that can cause disease [10].

There is significant geographic variation in the prevalence and type of ARGs in microbiota, implying that there may be environmental and lifestyle factors affecting the prevalence of ARGs. Hu and colleagues reported regional differences in ARG abundance; Chinese individuals have the greatest number of ARGs, followed by Danish and Spanish counterparts [11].

Southeast and South Asian countries are considered epicentres of antibiotic resistant bacteria [12] with high prevalence of extended spectrum beta- lactamase (ESBL) producing *Enterobacteriaceae*. In particular, the prevalence of ESBL *E. coli* has been reported to be as high as 50% in the Vietnamese general population [13], and even higher among infants hospitalized in a neonatal intensive care unit in Taiwan [14, 15]. In a recent study of healthy infants from Bangladesh, 82% had stool samples containing third generation cephalosporin-resistant *E. coli*, the majority of which were multidrug-resistant and ESBL-producers [5].

Despite the high prevalence of drug-resistant bacteria in community and hospital settings in Southeast Asia, most literature to date has focused on selected bacteria such as *E. coli* [16]. This is the first study in the region describing changes in the total antibiotic resistance gene (ARG) profile (also known as the resistome) of healthy infants over time. We aimed to evaluate changes in ARGs over the first year of life in the gut microbiome of infants from the Growing Up in Singapore Towards Healthy Outcomes (GUSTO) study, the largest longitudinal birth cohort study including pregnant women and their infants in Singapore. We also aimed to evaluate the role of demographic and social factors on development of the infant gut resistome.

## Methods

### Study population

**Fig. 1:**
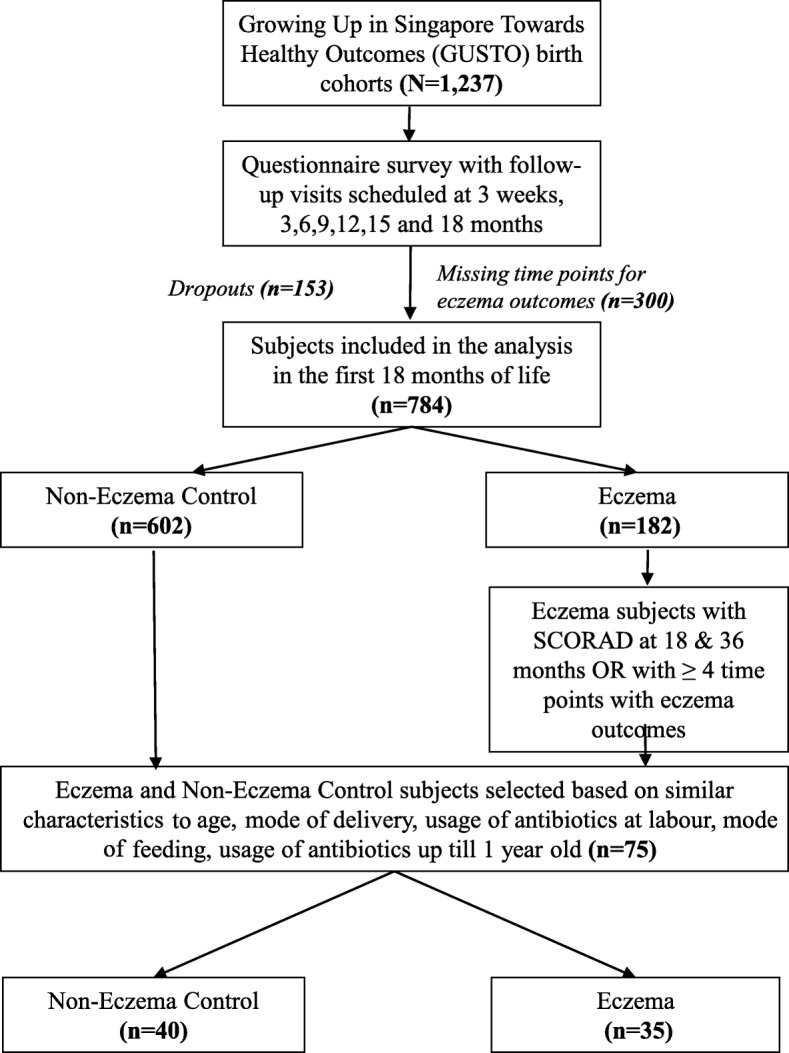
Flow chart of subject selection

The study cohort and methodology of the GUSTO study has been described in detail previously [17]. Briefly, we recruited pregnant mothers aged 18 and above, attending their first trimester antenatal dating scan from the two major maternity units in Singapore who agreed to enrol their offspring for future longitudinal follow-up. Mothers receiving chemotherapy, taking psychotrophic drugs or who had type 1 diabetes mellitus were excluded from the study. Information about subject demographics, family history of allergy, hospitalization, illnesses, social data and lifestyle factors was collected. Structured questionnaires were administered at 3 weeks, 3 months, 6 months and 12 months to collect information on each child’s health. Stool samples from enrolled infants were collected at 3 weeks (W3), 3 months (M3), 6 months (M6), and 12 months (M12). We analysed the presence of ARGs in the first year of life, in a subset of 75 infants at risk of eczema from the GUSTO birth cohort. The original aim of the study was to evaluate and compare stool microbiota in infants with eczema vs. those without eczema. Briefly, eczema subjects were selected based on parental report of physician-diagnosed eczema in the first 18 months of life and/or had SCORAD scores at 18 or 36 months [18]. Non-eczema subjects were matched with eczema subjects based on age, mode of delivery (caesarean/vaginal delivered), usage of antibiotics at labour (yes/no), mode of feeding in first 6 months of life (exclusively breastfeeding/partial breastfeeding/total formula feeding) and usage of antibiotics in the first year of life (yes/no). We included 40 non-eczema subjects and 35 eczema subjects. The study flowchart is presented in Fig. 1.

### Genomic DNA extraction

Genomic DNA was extracted from approximately 100–150 mg of stool from each specimen with the ZR Fecal DNA MicroPrep Kit (Zymo Research, USA) according to the manufacturer’s instructions.

### Metagenomic sequencing & bioinformatics analysis

For each stool sample, a sequencing library was first constructed using Illumina’s Truseq Nano DNA Library Preparation Kit (Illumina, San Diego, USA). The samples were sheared on a Covaris E220 to ~ 450 bp, following the manufacturer’s recommendation, and uniquely tagged with one of Illumina’s TruSeq HT DNA barcode combinations to enable sample pooling for sequencing.

The finished libraries were quantitated using Invitrogen’s Picogreen assay and the average library size was determined on Bioanalyzer 2100, using a DNA 7500 chip (Agilent). Library concentrations were then normalized to 4 nM and validated by qPCR on a ViiA-7 real-time thermocycler (Applied Biosystems), using qPCR primers recommended in Illumina’s qPCR protocol, and Illumina’s PhiX control library as standard. The libraries were then pooled at equimolar concentrations and sequenced on an Illumina HiSeq2500 sequencer in rapid mode at a read-length of 250 bp paired-end. Approximately 5Gb of sequencing data were obtained per sample so as to capture most of the novelty [19].

Genomic DNA sequences obtained from Illumina HiSeq paired-end sequencing were analyzed as follows: (i) Sequence quality check, (ii) Reads to protein alignment, and (iii) Taxonomical classification. To perform a quality check, reads shorter than 30 bp and low-quality sequences (<Q20 score) were removed. As elaborated by Illumina Inc. (2018), a sequencing quality score of 20 (Q20) represents an average error rate of 1 in 100 bases.

Therefore, removal of any sequence with a higher error rate than 1% (i.e., <Q20 score) allows for a corresponding call accuracy of at least 99%.

Thereafter, metagenomic reads were obtained by aligning the high-quality reads against human reference genome (hg19), whereby reads that were aligned to hg19 were discarded. The non-human metagenomic reads were then run on ResFinder 2.1. to check for the ARG’s conferring resistance to: aminoglycoside, tetracycline, beta-lactam, colistin, fosfomycin, fusidic acid, macrolide, nitroimidazole, oxazolidinone, phenicol, quinolone, rifampicin, sulphonamide, trimethoprim, and glycopeptide antibiotics.

Lastly, a lowest common ancestor (LCA)-based Taxonomical Classification of the aligned sequence reads was carried out on MEGAN6 (MEtaGenome Analyzer 6) using a bitscore cut-off of 100. A simple algorithm is utilized by MEGAN to assign each read to the LCA of the set of taxa that it hits in the comparison, whereby species-specific sequence reads will be assigned to the species taxon, while widely conserved sequence reads will be assigned to the high-order taxa [20].

### Statistical analysis

Univariate analysis using Pearson chi-square test was performed to assess associations between ARGs and demographic, lifestyle and clinical factors.

From the absolute number of normalized reads obtained from the LCA based Taxonomical Classification on MEGAN6, the relative abundance of each bacterial phylum, family, genus and species were calculated. Shannon and Simpson’s Diversity Indices were then calculated using relative abundance obtained at the species taxon.

For all statistical analysis, IBM SPSS Statistics (Version 24.0) was used (IBM Corporation, New York, USA), with two-tailed statistical tests and confidence intervals of 95% (i.e., *p*-value < 0.05). All graphical figures were plotted using Microsoft Excel 2017 (Microsoft Corporation, Washington, USA) and GraphPad Prism Version 7 (GraphPad Software, California USA).

## Results

### Study population and clinical characteristics

The majority of the subjects included in this study were healthy full-term infants (72/75, 96.0%), while 2 were delivered at 36 weeks and 1 at 35 weeks. Out of these 75 infants, 33.3% were delivered by caesarean delivery (Table 1). The proportion of female and male infants were similar and the majority were of Chinese ethnicity [42 (56%)]. Antibiotics were taken by 35 (46.7%) mothers during pregnancy and/or labour. Out of the 14 mothers that took antibiotics during pregnancy, 3 took it during the first trimester, 6 during second trimester and 5 during third trimester. A minority of the infants [16 (21.3%)] were prescribed antibiotics during the first year of life. The subjects included in this study were similar to the larger GUSTO cohort in all characteristics except consumption of antibiotics, illness and hospitalisation (Additional Table 1, Supplementary Data).

**Table 1:** Baseline demographic and clinical characteristics of subjects

Baseline Demographics	No. of subjects (*n* = 75)
	*n* (%)
**Gender**	
*Male*	38 (50.7%)
*Female*	37 (49.3%)
**Presence of siblings**	46 (61.3%)
**Mode of delivery**	
*Caesarean*	25 (33.3%)
*Vaginal*	50 (66.7%)
**Gestational age**	
*Term (≥37 weeks)*	72 (96.0%)
*Preterm*	3 (4.0%)
**Ethnicity**	
*Chinese*	42 (56%)
*Malay*	21 (28%)
*Indian*	12 (16%)
**Household monthly income**	
0-$999	2 (2.8%)
$1000–$1999	9 (12.7%)
$2000–$3999	25 (35.2%)
$4000–$5999	13 (18.3%)
> $6000	22 (31%)
**Maternal Tertiary Education**	46 (61.3%)
**Antibiotics during pregnancy and/or labour**	35 (46.7%)
**Class of antibiotics used during pregnancy and/ or labour**	
*Beta-lactam*	30 (40%)
*Macrolide, beta-lactam*	1 (1.3%)
*Macrolide*	3 (4.0%)
*Lincosamide*	1 (1.3%)
**Postnatal antibiotics within first 12 months**	16 (21.3%)
**Childcare attendance in first year**	7 (14%)
**Pet ownership at 12 months**	3 (6%)
**Smoking exposure in first year**	1 (1.3%)
**Maternal history of atopy**	18 (24%)
**Paternal history of atopy**	20 (26.7%)
**Eczema up to 18 months**	35 (46.7%)
**Rhinitis up to 18 months**	21 (28.4%)
**Wheeze with nebulizer use up to 18 months**	5 (7%)
**Allergen sensitization at 18 months**	15 (23.1%)
**Hospitalisation in the first year**	5 (6.7%)
**Illness diagnosed in the first year**	38 (50.7%)

### Antibiotic resistance gene profile

**Fig. 2:**
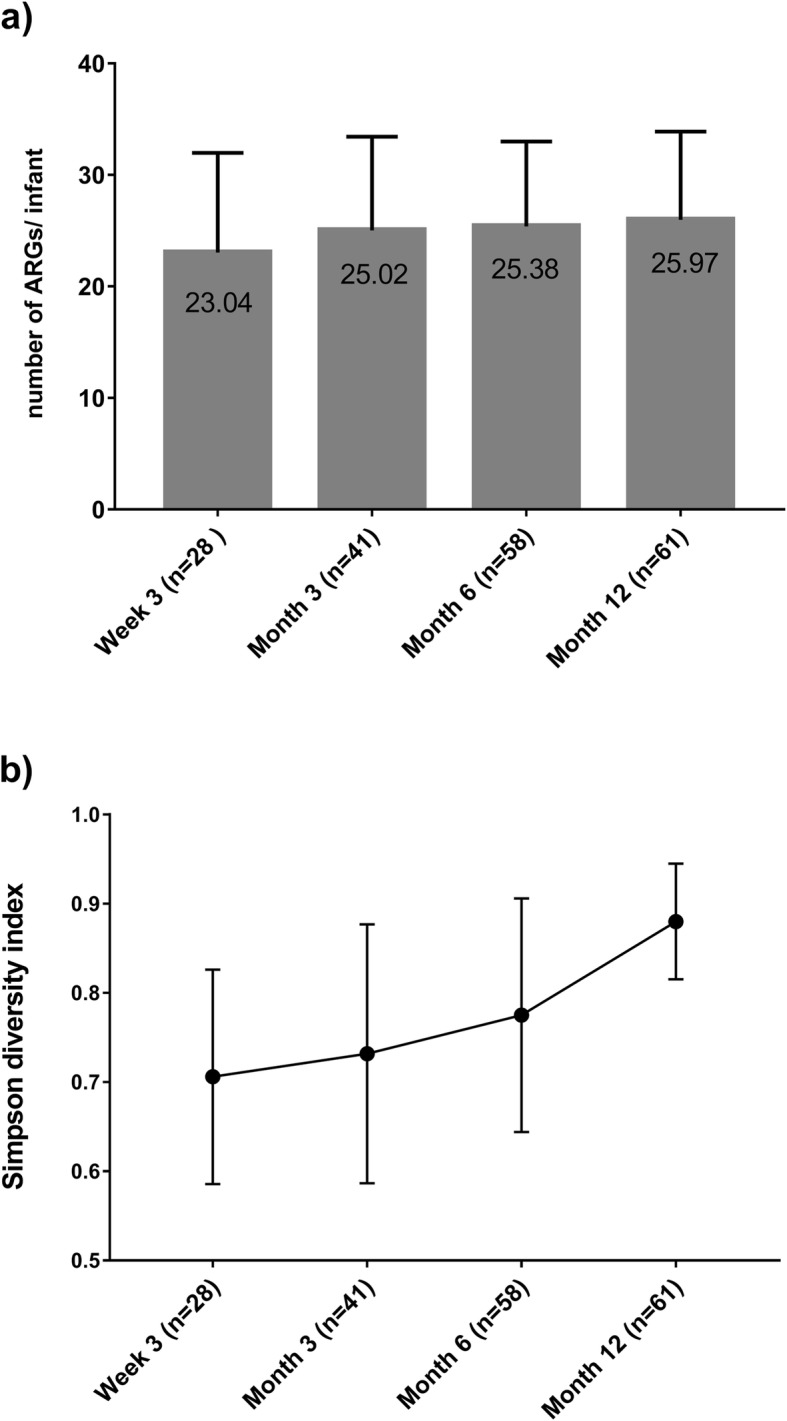
**a** Mean number of ARGs per infant over time. **b.** Microbial diversity of stool samples over time as determined by Simpsons Diversity Index

There were 188 available stool samples from 75 infants; 28 at week 3, 41 at month 3, 58 at month 6 and 61 at month 12. Eight (10.7%) subjects had stool samples available at all 4 timepoints. There were 228 unique ARGs detected among all samples. The mean number of ARGs per infant increased with age (23.0 at W3, 25.0 at M3, 25.4 at M6 and 26.0 at M12, *p* < 0.05, Fig. 2a) Microbial diversity of the stool samples also increased over time, as measured by the Shannon diversity index (Fig. 2b).

The most common ARGs identified confer resistance to aminoglycoside, beta-lactam, macrolide and tetracycline antibiotics; all infants harboured these ARGs at some point in the first year of life (Additional Figure 1, Supplementary Data). Overall, the most common ARGs were fosA, blaZ, tet (M) and mef (A), conferring fosfomycin, beta-lactam, tetracycline and macrolide resistance, respectively (Table 2). The most common ARGs detected varied over time. At week 3, the most prevalent ARGs were fosA and blaZ which were present in more than 95% of infants, while at month 12 the most prevalent ARG was mefA, found in 93.4% of infants (Table 2).

**Table 2:** Most commonly detected antibiotic resistance genes and associated antibiotic resistance phenotypes by time point

Antibiotic resistance profile and associated antibiotic resistance genes		Timepoint			
		W3 (*n* = 28) No. (%)	M3 (*n* = 41) No. (%)	M6 (*n* = 58) No. (%)	M12 (*n* = 61) No. (%)
**Fosfomycin**	**fosA**	27 (96.43%)	34 (82.93%)	47 (81.03%)	37 (60.66%)
**Beta-lactam**	**blaZ**	27 (96.43%)	16 (39.02%)	12 (20.69%)	8 (13.11%)
**Tetracycline**	**tet(M)**	20 (71.43%)	26 (63.41%)	35 (60.34%)	38 (62.30%)
**Macrolide**	**mef(A)**	19 (67.86%)	31 (75.61%)	46 (79.31%)	57 (93.44%)
**Quinolone**	**oqxB**	19 (67.86%)	24 (58.54%)	23 (39.66%)	14 (22.95%)
**Quinolone**	**msr(D)**	18 (64.29%)	28 (68.29%)	47 (81.03%)	53 (86.89%)
**Macrolide**	**erm(B)**	17 (60.71%)	26 (63.41%)	48 (82.76%)	54 (88.52%)
**Macrolide**	**lsa(A)**	17 (60.71%)	32 (78.05%)	29 (50%)	5 (8.20%)
**Tetracycline**	**tet(W)**	15 (53.57%)	31 (75.61%)	51 (87.93%)	50 (81.97%)
**Aminoglycoside**	**aac(6′)-aph(2″)**	15 (53.57%)	10 (24.39%)	21 (36.21%)	34 (55.74%)

The number and percentage of subjects with the antibiotic resistance genes are shown

### Association between usage of beta-lactam class of antibiotics during pregnancy and/or labour and acquisition of beta-lactam resistance genes in the offspring

Among the 35 women receiving antibiotics during pregnancy and/or labour, beta-lactam antibiotics were the most commonly given antibiotic class [30/35 (85.7%)] (Table 1). Details of the antibiotics used are presented in Additional Tables 2 and 3, Supplementary Data.

Given the high usage of the beta-lactam class of antibiotics during pregnancy and/or labour, we next determined if this affected the acquisition of beta-lactam resistance genes in the offspring. All ARGs that conferred beta-lactam resistance were analysed. Offspring of mothers exposed to beta-lactam antibiotics during pregnancy and/or labour had significantly increased detection of the beta-lactam-resistance conferring genes, cepA-44 (9/80, 11.3%) and cfxA (14/80, 17.5%), in their stool samples compared to offspring of mothers who did not take these antibiotics (1/101, 1.0%; 6/101, 5.9% respectively *p* < 0.01).

There were no significant associations between the use of beta-lactam antibiotics during pregnancy and/or labour and other ARGs conferring beta-lactam resistance in the first year of life. The presence of specific ARGs were not significantly associated with gender, race, delivery mode, postnatal antibiotics use and eczema diagnosis in infancy.

### Beta-lactamase type

We next characterized the type of beta-lactamase genes; the majority were ESBLs [CTX-M beta lactamases (class A), OXA beta-lactamases (class D), SHV beta-lactamases (class A) and TEM beta-lactamases (class A), Table 3]. The most common ESBL genes was blaTEM-1B, present in 10 (35.7%) subjects at week 3, 22 subjects (53.7%) at month 3, 34 subjects (58.6%) at month 6, and 29 subjects (47.5%) at month 12. Carbapenemase detection was rare and only CMY (class C) carbapenemases were detected. Among stool samples at each time point, carbapenemase genes were found in 1 (3.6%) at W3, 4 (9.8%) at M3, 7 (12.0%) at month 6 and 4 (6.6%) at month 12. Most carbapenemase genes (blaCMY-34, blaCMY-49, blaCMY-83, blaCMY-98) were detected at 1 timepoint only for a given infant, suggesting that they do not persist with time. blaCMY-2 was detected in multiple infants including 1 subject at week 3, 2 at month 3, 4 at month 6 and 2 at month 12. NDM and KPC type carbapenemases were not detected in the infants.

**Table 3:** Detected beta-lactamase genes

Beta-lactam resistance genes		W3 *n* = 28	M3 *n* = 41	M6 *n* = 58	M12 *n* = 61
Carbapenemases					
CMY (Class C)	blaCMY-2	1	2	4	2
	blaCMY-34	0	0	0	1
	blaCMY-49	0	1	0	0
	blaCMY-76	0	0	2	1
	blaCMY-83	0	0	1	0
	blaCMY-98	0	1	0	0
Extended spectrum beta-lactamases					
CTX-M beta lactamases (Class A)	blaCTX-M-14	0	1	1	0
	blaCTX-M-15	0	1	0	1
	blaCTX-M-27	0	1	1	0
	blaCTX-M-40	0	0	1	1
	blaCTX-M-78	0	0	0	1
	blaCTX-M-8	0	0	2	1
OXA beta-lactamases (class D)	blaOXA-1	0	0	1	2
	blaOXA-10	0	1	0	0
	blaOXA-116	1	0	0	0
	blaOXA-120	0	0	1	0
	blaOXA-184	0	0	0	1
	blaOXA-347	2	9	11	14
	blaOXA-85	1	0	1	0
SHV beta-lactamases (Class A)	blaSHV-1	9	9	7	3
	blaSHV-108	0	0	1	0
	blaSHV-11	5	8	7	4
	blaSHV-142	1	0	0	0
	blaSHV-26	1	0	0	0
	blaSHV-27	0	0	0	4
	blaSHV-28	1	0	0	0
	blaSHV-38	0	0	1	0
	blaSHV-63	0	0	0	1
	blaSHV-75	0	0	1	0
	blaSHV-83	1	2	0	0
	blaSHV-85	0	0	0	1
	blaSHV-93	0	1	1	0
TEM beta-lactamases (Class A)	blaTEM-176	1	1	1	2
	blaTEM-1B	10	22	34	29
	blaTEM-1C	0	0	0	3
	blaTEM-1D	0	0	0	1

The number of subjects with beta lactam resistance genes detected in their stool samples at each time point are shown

### Dynamics of ARGs over time

We next analysed the most commonly-detected ARGs from early life (3 weeks) up to 12 months of age. The analysis was performed among 22 infants who had stool samples at both week 3 (first timepoint) and month 12 (last timepoint). Among these infants, there were 3 ARGs that were present in both samples. These ARGs were mef(A) conferring macrolide resistance, aadE conferring aminoglycoside resistance, and msr(D) which conferred macrolide, lincosamide and streptogramin B resistance (Table 4). Among infants who harboured ESBL genes at week 3, we found blaTEM-1b present at month 12 in 6/9 (67%) subjects and blaOXA-347 in 2/2 (100%) subjects (Table 5).

**Table 4:** Presence of antibiotic resistance genes (ARGs) at time points during the first year of life

		Number of subjects#		% of subjects with ARG present at time points	Number of subjects*				% of subjects with ARG present at time points		
Antibiotic resistance genotype	ARG	W3	W3 and M12	W3 and M12	W3	W3 to M3	W3 to M6	W3 to M12	W3 to M3	W3 to M6	W3 to M12
Fosfomycin	fosA	21	11	52%	8	7	6	4	88%	75%	50%
Beta-lactam	blaZ	21	2	10%	7	1	1	0	14%	14%	0%
Aminoglycoside	aac(6′)-aph(2″)	11	5	45%	5	1	0	0	20%	0%	0%
Macrolide	lsa(A)	13	2	15%	5	5	3	1	100%	60%	20%
Quinolone	oqxB	14	4	29%	5	4	1	1	80%	20%	20%
Macrolide	erm(B)	13	11	85%	4	2	2	2	50%	50%	50%
Macrolide	erm(X)	6	5	83%	4	3	2	1	75%	50%	25%
Macrolide	mef(A)	15	15	100%	4	4	3	3	100%	75%	75%
Macrolide, Lincosamide and Streptogramin B	msr(D)	14	14	100%	4	4	2	2	100%	50%	50%
Tetracycline	tet(M)	15	11	73%	4	2	1	1	50%	25%	25%
Aminoglycoside	aph(3′)-III	7	5	71%	3	2	1	1	67%	33%	33%
Sulphonamide	sul2	8	4	50%	3	3	2	2	100%	67%	67%
Tetracycline	tet(O)	6	4	67%	3	3	3	2	100%	100%	67%
Tetracycline	tet(Q)	5	4	80%	3	3	3	3	100%	100%	100%
Tetracycline	tet(W)	11	10	91%	3	3	3	3	100%	100%	100%
Beta-lactam	blaTEM-1b	9	6	67%	2	2	2	1	100%	100%	50%
Aminoglycoside	aadE	1	1	100%	1	1	0	0	100%	0%	0%

# subjects with stool samples at both week 3 and month 12 (*N* = 22). *subjects with stool samples at all timepoints (*N* = 8)

**Table 5:** Presence of extended spectrum beta-lactamase (ESBL) resistance genes in the first year of life

	Number of subjects#		Presence	Number of subjects*				Presence		
Extended Spectrum Beta-lactamases	W3	W3 and M12	W3 and M12	W3	W3 to M3	W3 to M6	W3 to M12	W3 to M3	W3 to M6	W3 to M12
blaTEM-1b	9	6	67%	2	2	2	1	100%	100%	50%
blaSHV-1	8	1	13%	2	0	0	0	0%	0%	0%
blaSHV-11	3	0	33%	1	1	0	0	100%	0%	0%
blaSHV-142	1	0	0%	1	0	0	0	0%	0%	0%
blaSHV-83	1	0	0%	1	0	0	0	0%	0%	0%
blaOXA-116	1	0	0%	1	0	0	0	0%	0%	0%
blaOXA-347	2	2	100%	1	1	1	1	100%	100%	100%

# subjects with stool samples at both week 3 and month 12 (*N* = 22) *subjects with stool samples at all timepoints (*N* = 8)

An additional subgroup analysis was performed among infants who had stool samples collected at all timepoints. Among the 8 infants who had stool samples analysed at all 4 timepoints, there were 9 ARGs that were present at both week 3 and month 3 samples. These ARGs were lsa(A), mef(A), msr(D), sul2, tet(O), tet(Q), tet(W), blaTEM-1B and aadE which conferred macrolide, macrolide, sulphonamide, tetracycline, beta-lactam, aminoglycoside, macrolide, lincosamide and streptogramin B resistance respectively. There were 4 ARGs that were present at all timepoints from week 3 to month 6 and they were tet(O), tet(Q), tet(W), blaTEM-1B, which confer tetracycline and beta-lactam resistance. There were only 2 ARGs that were present at all timepoints from week 3 to month 12: tet(Q) from *Bacteroides* and tet(W) from *Bifidobacterium*, both conferring tetracycline resistance (Table 4).

### Beta-lactam resistant *E. coli* and *K. pneumoniae*

Beta-lactam resistant *E. coli,* which is endemic in Singapore, was detected in 4 (5.3%) subjects at different timepoints, including, 1 subject at week 3, 2 at month 3 and 2 at month 6 respectively. No beta-lactam resistant *E. coli* was detected at month 12. There were 3 ESBL genes detected in *E. coli*, namely blaCMY-2, blaCTX-M15 and blaTEM-1b (Table 6). In contrast, beta-lactam resistant *K. pneumoniae* was more common and was detected in 32 (42.7%) subjects at any time point. The prevalence of beta-lactam resistant *K. pneumoniae* declined over time; it was found in 13 subjects at week 3, 10 at month 3, 11 at month 6, 8 at month 12. Among these *K. pneumoniae*, there were 9 ESBLs detected, all of which were SHV beta-lactamases (class A) (Table 6).

**Table 6:** Extended spectrum beta-lactamase genes found in *E. coli* and *Klebsiella pneumoniae*

Extended-Spectrum Beta-lactam resistance genes		Species	W3	M3	M6	M12
CMY (Class C)	blaCMY-2	*Escherichia coli*	1	1	1	0
CTX-M beta lactamases (Class A)	blaCTX-M15	*Escherichia coli*	0	1	0	0
SHV beta-lactamases (Class A)	blaSHV-1	*Klebsiella pneumoniae*	6	7	4	2
	blaSHV-108	*Klebsiella pneumoniae*	0	0	1	0
	blaSHV-11	*Klebsiella pneumoniae*	5	2	4	3
	blaSHV-142	*Klebsiella pneumoniae*	1	0	0	0
	blaSHV-26	*Klebsiella pneumoniae*	1	0	0	0
	blaSHV-27	*Klebsiella pneumoniae*	0	0	0	2
	blaSHV-38	*Klebsiella pneumoniae*	0	0	1	0
	blaSHV-85	*Klebsiella pneumoniae*	0	0	0	1
	blaSHV-93	*Klebsiella pneumoniae*	0	1	1	0
TEM beta-lactamases (Class A)	blaTEM-1b	*Escherichia coli*	0	0	1	0

## Discussion

We report the first longitudinal cohort study in Southeast Asia to evaluate the diversity and dynamics of the infant gut resistome over the first year of life. We observed that in an area with high endemic bacterial drug resistance, the infant gut harbours a wide diversity of ARGs from early in infancy [4, 6–9]. We also observed significant differences among the ARGs prevalent in early infancy compared to those prevalent at 1 year of life. Generally, the number of ARGs per infant stool specimen increased over time during the first year of life. This increasing prevalence may be due to environmental acquisition of some genes or the emergence of resistance among normal flora. A minority of ARGs persisted through the first year of life, demonstrating a highly dynamic infant resistome [21, 22].

Our findings are supported by studies from other parts of the world. Gosalbes and colleagues found a high prevalence of resistance to beta-lactam antibiotics and tetracycline in both meconium and stool samples from 1-week-old infants, suggesting the acquisition of the infant gut resistance reservoir even before birth [22]. A functional screen for ARGs in the gut microbiota of 22 healthy 6-month old infants from Ireland, who had not received any prior antibiotics, demonstrated the presence of a variety of genes encoding resistance to aminoglycosides and beta-lactams [23]. Nogacka and colleagues also found a higher occurrence of some beta-lactamase encoding genes in vaginally-delivered term Spanish infants whose mothers received intra-partum antimicrobial prophylaxis, compared to those whose mothers did not receive intra-partum antibiotics [24]. Wintersdorff et al. also noted increasing prevalence of ARGs over time in infants, in Germany [25].

The prevalence of ARGs encoding resistance to antibiotics that are rarely used in humans, such as chloramphenicol, may imply selection pressure from the environment, or food and agriculture, possibly via maternal diet. If so, this is likely to vary across different regions throughout the world. Early in infancy, certain ARGs may be easily acquired from breast milk or even environmental contaminants of formula milk [26, 27]. Later on, as infants are weaned and begin eating table foods, ARGs may be acquired from ingesting meat and vegetables. It is known that antibiotics are often used in livestock for growth promotion and disease prevention, particularly in settings of intensive animal production [28]. This has been known to be implicated in the development of antimicrobial-resistant bacteria that can be spread to humans. The tendency to acquire or lose ARGs may be additionally affected by a complex interaction of epigenetic factors, but the large diversity of ARGs may make it challenging to find significant associations between specific ARGs and the various clinical variables studied.

We observed that maternal beta-lactam exposure during pregnancy or delivery was associated with the detection of beta-lactam resistance genes among infants. This is consistent with the work by Nogacka et al. [24], who found that intra-partum antibiotics affect the composition of the infant gut microbiome.

Surprisingly, we did not observe any association between the mode of delivery and acquisition of ARGs in the offspring. This finding differs from a previous study which showed that tetracycline resistance genes such as tet(M), tet(O), tet(W), and tet(Q) were identified in maternal vaginal flora, and that their transmission to offspring was dependent on mode of delivery; 50% of the infants delivered vaginally harboured tetracycline resistance genes tet(M) and tet(O) found in mothers, while 16 and 13% of infants delivered by C-section had tet(O) and tet(W) respectively [9].

The prevalence of ARGs in the gut resistome did not always correlate with common multidrug resistance phenotypes in the community. For example, mecA, which confers resistance to all beta-lactam antibiotics and is found in most methicillin-resistant *Staphylococcus aureus* (MRSA) strains, was not prevalent in the infant resistome despite its high incidence in adults in the hospital and community [29]. Similarly, beta-lactam-resistant *E. coli*, which is endemic in Singapore, was only found in 5% of the infants. In contrast, beta-lactam-resistant *K. pneumoniae,* which is also common in the community, was found in 42% of the infants.

In our study, we also found several ESBL genes in infants’ stools in the first year of life, independent of the selection pressure of antibiotic exposure. This highlights the endemicity of these strains in Singapore, and the global problem of transmission of multi-drug antibiotic resistant bacteria. Surprisingly few infants harboured drug-resistant *E. coli*, despite widespread dissemination of drug-resistant *E. coli* sequence types in Singapore [30–33]. In contrast, drug-resistant *K. pneumoniae* was detected in a number of infants from the GUSTO cohort. *K. pneumoniae* is known to acquire ARGs via de novo mutations, plasmid dissemination and transfer of ARGs. This underscores the potential for the infant gut resistome to facilitate the rise of extremely drug resistant (XDR) strains carrying ‘super resistomes’ [34].

The infant gut microbiome in dysbiosis has been implicated in diseases such as necrotizing enterocolitis and allergic disease. However, the significance of the infant gut resistome specifically and its role in human disease is still unclear. It is not known what constitutes a “healthy” resistome and the implications of ARG diversity in the infant gut. It may be a reflection of our antimicrobial use not just in pregnancy but also in the food industry. It may have longer lasting implications on the nature and severity of bacterial infections acquired later on in life. Potentially, the infant gut resistome can influence the incidence of multi-drug resistant (MDR) infections, by harbouring ARGs in commensals that transfer to pathogenic bacteria [10]. Better understanding of the naïve infant gut resistome may help to build the evidence base for antimicrobial stewardship.

The strengths of this study are the longitudinal collection of infants’ stool specimens and follow up of the infants by structured questionnaires in early life. In addition, the large sample size and higher endemic resistance rates in Singapore are also strengths compared to earlier studies. However, a limitation of this study is that maternal stool specimens and infant stool specimens at birth were not available. It is hence not possible to identify which ARGs were likely to have been vertically transmitted. In addition, not all infants had stool samples available at all time points, thereby limiting analyses of ARG persistence over time. The large diversity of ARGs may also make it challenging to find clinically significant associations between specific ARGs and demographic and clinical variables. Our studied population also overrepresents subjects with eczema, limiting its generalizability as they may carry a higher risk of ARG carriage than the baseline population.

## Conclusion

In conclusion, this study describes the diversity and dynamic nature of the infant gut resistome in a region with high endemic resistance rates, and the paucity of known variables that affect the diversity of the gut resistome. Future work should focus on the mode of acquisition, transmission, and persistence of these ARGs. Metagenomic comparisons between microbiota in infants and their adult household contacts would also help elucidate factors influencing the establishment of the infant gut resistome.

## Supplementary information

**Additional file 1: Table S1.** Comparison of demographic variables between subjects included and excluded from study. **Figure S1.** Antibiotic resistance genotypes over time. The percentage of subjects with the antibiotic resistance genotype are presented over time. **Table S2.** Maternal antibiotic exposures during pregnancy. **Table S3.** Maternal antibiotic exposures during labour.

## Data Availability

The datasets used and/or analysed during the current study are available from the corresponding author on reasonable request.

## Abbreviations

ARGs: Antibiotic resistance genes
ESBL: Extended spectrum beta- lactamases.
GUSTO: Growing Up in Singapore Towards Healthy Outcomes
