# Supplementary material for: Longitudinal assessment of antibiotic resistance gene profiles in gut microbiomes of infants at risk of eczema
Source: BMC Infect Dis. 2020 Apr 28;20:312. doi: 10.1186/s12879-020-05000-y (PMC7189448; doi:10.1186/s12879-020-05000-y)
Supplement: Supplementary file 1 — Additional file 1: Table S1. Comparison of demographic variables between subjects included and excluded from study. Figure S1. Antibiotic resistance genotypes over time. The percentage of subjects with the antibiotic resistance genotype are presented over time. Table S2. Maternal antibiotic exposures during pregnancy. Table S3. Maternal antibiotic exposures during labour. [file 12879_2020_5000_MOESM1_ESM.zip › Cleaned_ARGpaper_ Supplementary Data final EL 110320R2.docx]

**Additional Figures and Tables**

**Additional Table 1.** Comparison of demographic variables between subjects included and excluded from study.

| **Baseline Demographics** | No. of subjects (n=75) | excluded (n=1162) |  |
| --- | --- | --- | --- |
|  | n (%) | n (%) | p value |
| **Gender** |  |  |  |
| *Male* | 38 (50.7%) | 581 (53%) | 0.69 |
| *Female* | 37 (49.3%) | 515 (47%) |  |
| **Presence of siblings** | 46 (61.3%) | 589 (53.8%) | 0.21 |
| **Mode of delivery** |  |  |  |
| *Caesarean* | 25 (33.3%) | 322 (29.4%) | 0.47 |
| *Vaginal* | 50 (66.7%) | 772 (70.6%) |  |
| **Gestational age** |  |  |  |
| *Term (≥37 weeks)* | 72 (96.0%) | 992 (90.7%) | 0.12 |
| *Preterm* | 3 (4.0%) | 102 (9.3%) |  |
| **Ethnicity** |  |  |  |
| *Chinese* | 42 (56%) | 648 (55.8%) | 0.89 |
| *Malay* | 21 (28%) | 303 (26.1%) |  |
| *Indian* | 12 (16%) | 211 (18.2%) |  |
| **Household monthly income** |  |  |  |
| 0-$999 | 2 (2.8%) | 27 (23.8%) | 0.70 |
| $1000-$1999 | 9 (12.7%) | 143 (12.3%) |  |
| $2000-$3999 | 25 (35.2%) | 325 (28%) |  |
| $4000-$5999 | 13 (18.3%) | 277 (23.8%) |  |
| >$6000 | 22 (31%) | 309 (26.6%) |  |
| **Maternal Tertiary Education** | 46 (61.3%) | 668 (58.4%) | 0.62 |
|  |  |  |  |
| **Antibiotics during pregnancy and/or labour** | 35 (46.7%) | 466 (45.2%) | 0.49 |
| **Postnatal antibiotics within first 12 months** | 16 (21.3%) | 387 (47.4%) | <0.001 |
|  |  |  |  |
| **Childcare attendance in first year** | 7 (14%) | 69 (15.5%) | 0.78 |
| **Pet ownership at 12months** | 3 (6%) | 51 (10.2%) | 0.34 |
| **Smoking exposure in first year** | 1 (1.3%) | 43 (5%) | 0.16 |
|  |  |  |  |
| **Maternal history of atopy** | 18 (24%) | 191 (23.2%) | 0.77 |
| **Paternal history of atopy** | 20 (26.7%) | 180 (21.8%) | 0.27 |
| **Eczema up to 18 months** | 35 (46.7%) | 148 (12.7%) | <0.001 |
| **Rhinitis up to 18 months** | 21 (28.4%) | 411 (53.9%) | <0.001 |
| **Wheeze with nebulizer use up to 18 months** | 5 (7%) | 101 (15.1%) | 0.06 |
| **Allergen sensitization at 18 months** | 15 (23.1%) | 99 (12.6%) | 0.02 |
|  |  |  |  |
| **Hospitalisation in the first year** | 5 (6.7%) | 178 (22.7%) | 0.001 |
| **Illness diagnosed in the first year** | 38 (50.7%) | 523 (63.5%) | 0.027 |

**Additional Figure 1.** Antibiotic resistance genotypes over time. The percentage of subjects with the antibiotic resistance genotype are presented over time.

| **Class** | **Drug Class** | **Type of antibiotics received during pregnancy** | **No. of subjects** |
| --- | --- | --- | --- |
| **Beta-lactam** | Beta-lactam | Cefalexin capsule | 3 |
| **Beta-lactam** | Penicillin | Amoxicillin capsule | 6 |
| **Beta-lactam** | Penicillin | Amoxicillin capsule, Cloxacillin capsule | 1 |
| **Macrolide, Beta-lactam** | Macrolide, Penicillin | Erythromycin ESS, Amoxicillin | 3 |
| **Macrolide** | Macrolide | Azithromycin | 1 |

**Additional Table 2.** Maternal antibiotic exposures during pregnancy.

**Additional Table 3.** Maternal antibiotic exposures during labour.

| **Class** | **Drug Class** | **Type of antibiotics during labour** | **No of subjects** |
| --- | --- | --- | --- |
| **Lincosamide** | Lincosamide | Clindamycin | 1 |
| **Beta-lactam** | Penicillin | Penicillin G | 13 |
| **Beta-lactam** | Penicillin | Ampicillin/Amoxicillin | 7 |
| **Beta-lactam** | Penicillin, Cephalosporin | Penicillin G, Cefazolin | 1 |
| **Beta-lactam** | Cephalosporin | Cefazolin | 4 |
| **Beta-lactam** | Cephalosporin | Rocephine | 1 |
