# Supplementary figures and images for: Longitudinal assessment of antibiotic resistance gene profiles in gut microbiomes of infants at risk of eczema
Source: BMC Infect Dis. 2020 Apr 28;20:312. doi: 10.1186/s12879-020-05000-y (PMC7189448; doi:10.1186/s12879-020-05000-y)

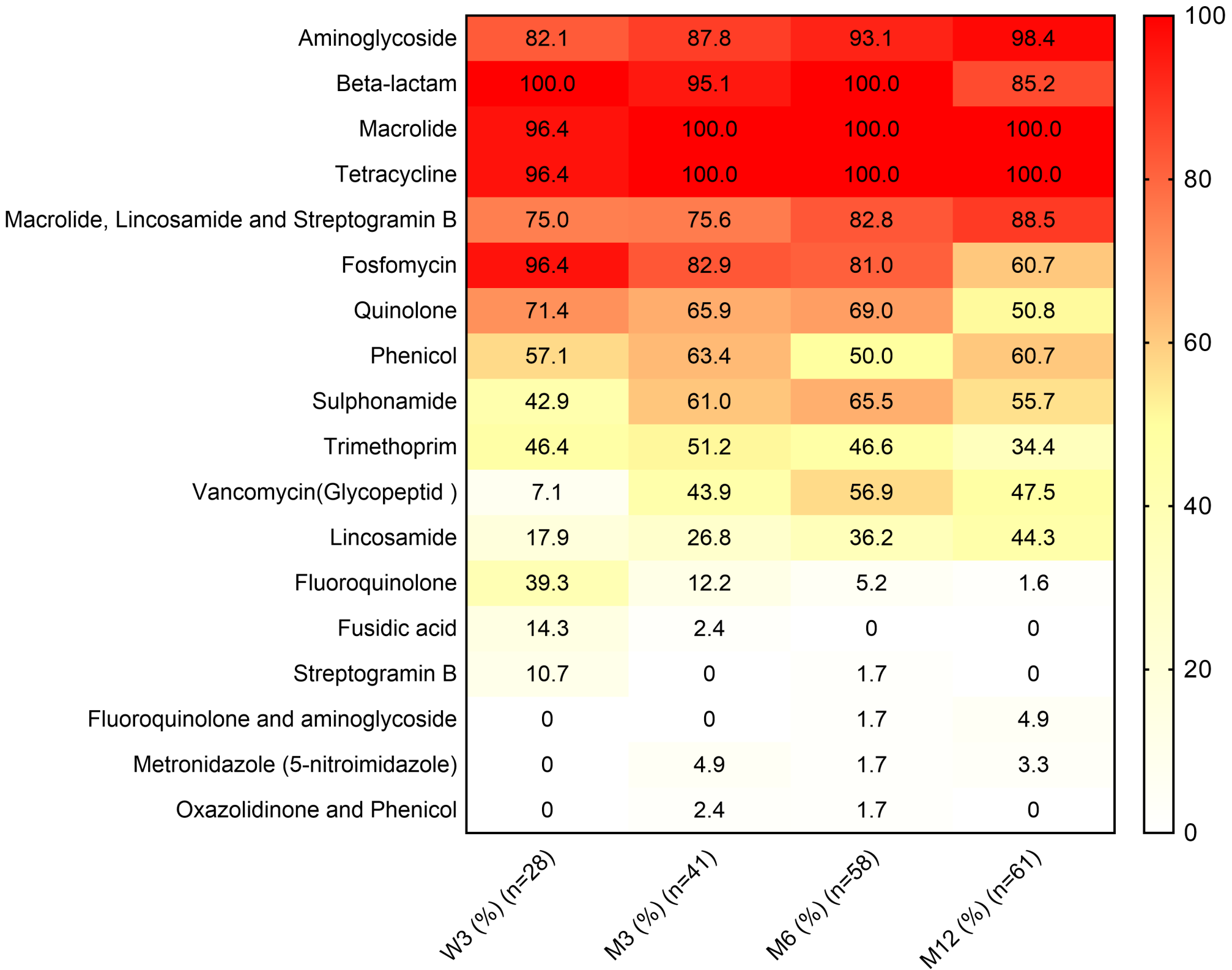

Supplement: Supplementary file 1 — Additional file 1: Table S1. Comparison of demographic variables between subjects included and excluded from study. Figure S1. Antibiotic resistance genotypes over time. The percentage of subjects with the antibiotic resistance genotype are presented over time. Table S2. Maternal antibiotic exposures during pregnancy. Table S3. Maternal antibiotic exposures during labour. [file 12879_2020_5000_MOESM1_ESM.zip › ARG paper Additional Figure 1 EL 110320R2.pdf]
